# Subsequent pregnancy outcomes among women with tubal ectopic pregnancy treated with methotrexate

**DOI:** 10.1530/RAF-23-0019

**Published:** 2023-06-15

**Authors:** Scott C Mackenzie, Catherine A Moakes, W Colin Duncan, Stephen Tong, Andrew W Horne

**Affiliations:** 1 MRC Centre for Reproductive Health, University of Edinburgh, Edinburgh, UK; 2 Birmingham Clinical Trials Unit, University of Birmingham, Birmingham, UK; 3 Department of Obstetrics and Gynaecology, University of Melbourne, Melbourne, Australia

**Keywords:** tubal ectopic pregnancy, methotrexate, pregnancy outcomes, recurrence, risk factor

## Abstract

**Graphical abstract:**

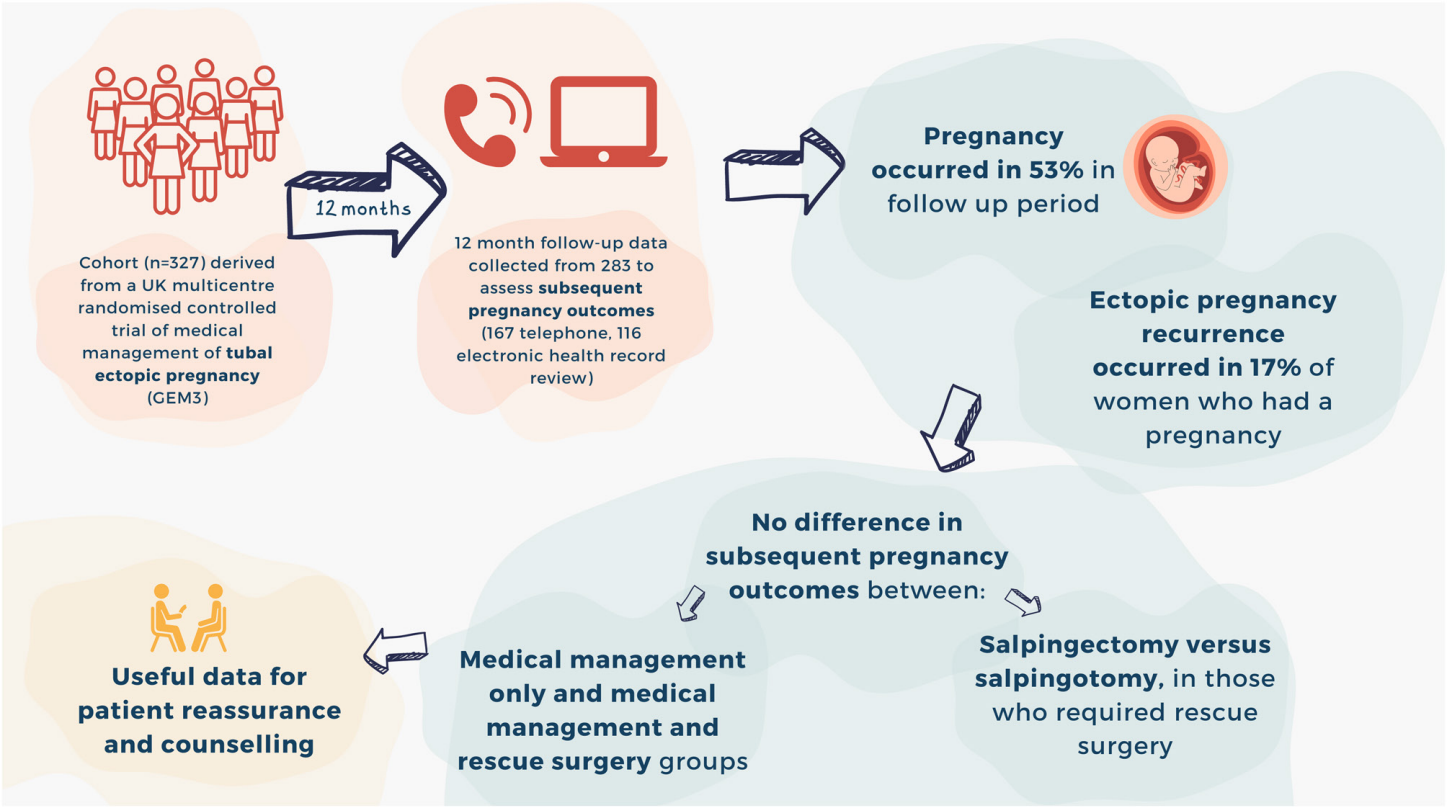

**Lay summary:**

An ectopic pregnancy occurs when an embryo implants outside of the uterus, usually in a fallopian tube. When detected early, treatment is often with a medication called methotrexate. When methotrexate does not work, surgery is required. A recent clinical trial of ectopic pregnancy treatment (called GEM3) found that adding a drug called gefitinib to methotrexate did not reduce the need for surgery. We have used data from the GEM3 trial, combined with data collected 12 months after the trial finished, to investigate post-methotrexate pregnancy outcomes. We found no difference in pregnancy rates, pregnancy loss rates and recurrent ectopic pregnancy rates between those treated medically only and those who subsequently also needed surgery. The surgical technique used also did not affect pregnancy rates. This research provides reassurance that women with ectopic pregnancies treated medically who need surgery have similar post-treatment pregnancy outcomes to those treated successfully medically.

## Research letter

Pre-treatment counselling for women with unruptured tubal ectopic pregnancies considering their treatment options requires the provision of information on post-treatment pregnancy outcomes, including rates of ectopic pregnancy recurrence. Current UK NICE early pregnancy guidelines are based on low-quality evidence and estimate the long-term ectopic pregnancy recurrence rate at ~18.5% (NICE 2021). Further, they relay findings showing no differences in rates of subsequent pregnancy or ectopic pregnancy recurrence between management methods (de Bennetot *et al.* 2012, Fernandez *et al.* 2013, Park *et al.* 2017). Approximately 30% of women with a scan-diagnosed ectopic pregnancy actively managed with methotrexate experience treatment failure and require rescue surgery, and little is known about subsequent pregnancy outcomes in this group.

We describe subsequent pregnancy outcomes in women with tubal ectopic pregnancy managed with methotrexate using data from a UK multicentre randomised controlled trial comparing methotrexate and gefitinib vs methotrexate and placebo for the treatment of tubal ectopic pregnancy (GEM3: Horne *et al.* 2023). Trial participants were women with an ultrasound diagnosed definite (or probable) tubal ectopic pregnancy with pre-treatment serum hCG levels of ≥1000 IU/L and ≤5000 IU/L. The trial found adding gefitinib to methotrexate was not superior to placebo. After randomisation to treatment, trial participants were contacted at 12 months to provide subsequent pregnancy outcome data. Where telephone contact was unsuccessful, electronic health records were reviewed. Post-treatment pregnancy outcomes were summarised with descriptive statistics and the groups were compared using chi-squared tests.

Subsequent pregnancy outcome data were obtained for 283/327 trial participants (167 contacted by telephone; 116 from electronic health records). Follow-up data from both randomisation groups (methotrexate and gefitinib; methotrexate and placebo) were combined owing to no between-group differences in subsequent pregnancy outcomes (Supplementary Table 1, see section on supplementary materials given at the end of this article). Pregnancy occurred in 53% (149/283) of participants in the 12-month follow-up period. There was no difference in subsequent pregnancy rates between ‘medical management only’ and ‘medical management and rescue surgery’ groups (Table 1). Surgical approach to ectopic pregnancy treatment (salpingectomy vs salpingotomy) did not affect subsequent pregnancy rates. Among women who had a pregnancy within the follow-up period, a live birth occurred in 65% (93/142), any pregnancy loss occurred in 40% (55/136) and recurrent ectopic pregnancy occurred in 17% (22/131). No difference was observed in the rates of live birth, pregnancy loss or recurrent ectopic pregnancy between treatment groups or between those who had salpingectomy vs salpingotomy. Participant characteristics (body mass index, chlamydial infection history and smoking status) were investigated for association with recurrent ectopic pregnancy using univariate and multivariate log-binomial models. No significant associations were identified; however, this analysis was limited by the small sample of women in which ectopic pregnancy occurrence recurred. Similarly, the power to detect differences in subsequent pregnancy outcomes between treatment groups was limited owing to the small sample sizes in observed groups. Intention to conceive in the follow-up period was not recorded and we were therefore unable to stratify results accordingly.

**Table 1:** Pregnancy rates and subsequent pregnancy outcomes among women with tubal ectopic pregnancy treated medically. Data are presented as *n* or as *n* (%).

	MM + RS			MM only	*P*-value*
	Salpingectomy	Salpingotomy	All		
*n*	86	11	97	228	
Any pregnancy post-treatment	37 (51)	4 (50)	41 (51)	108 (53)	0.66
Missing	13	3	16	26	
Any live birth post-treatment^†^	20 (61)	3 (75)	23 (62)	70 (67)	0.62
Missing	4	0	4	3	
Any pregnancy loss post-treatment^†,‡^	16 (52)	1 (25)	17 (49)	38 (38)	0.26
Missing	6	0	6	7	
Any ectopic pregnancy post-treatment^†^	6 (22)	0 (-)	6 (19)	16 (16)	0.66
Missing	10	0	10	8	

*Medical management and rescue surgery vs medical management only; ^†^Only in women whom have had a pregnancy post-treatment; ^‡^Defined as miscarriage, ectopic pregnancy, stillbirth or molar pregnancy (excluding termination of pregnancy).

MM, medical management; RS, rescue surgery.

This prospective dataset strengthens current knowledge of the likelihood of ectopic pregnancy recurrence. Furthermore, it provides reassurance that women with tubal ectopic pregnancy who required rescue surgery following medical treatment have similar outcomes in their subsequent pregnancy as those with successful medical treatment alone. However, this study only reports on a 12-month follow-up period, which should be considered when interpreting the comparatively low post-treatment pregnancy rates (Fernandez *et al.* 2013).

## Supplementary Materials

Supplementary Table I. Pregnancy rates and subsequent pregnancy outcomes among women with tubal ectopic pregnancy medically, presented by GEM3 randomisation treatment (gefitinib and methotrexate versus placebo and methotrexate).

## Declaration of interest

AWH is a Co-Editor-in-Chief and WCD is an Associate Editor of Reproduction & Fertility. AWH and WCD were not involved in the review or editorial process for this paper, on which they are listed as authors. AWH has received honoraria for consultancy for Ferring, Roche Diagnostics, Nordic Pharma, Gesynta and Abbvie. WCD has received honoraria from Merck and Guerbet, and research funding from Galvani Biosciences. The other authors declare no competing interests.

## Funding

This project was supported by funding from the Efficacy and Mechanism Evaluation programmehttp://dx.doi.org/10.13039/501100001922, a Medical Research Councilhttp://dx.doi.org/10.13039/501100000265 and National Institute for Health Researchhttp://dx.doi.org/10.13039/100005622 partnership (grant reference number 14/150/03).

## Trial registration number

This study is a follow-up analysis of participants from the GEM3 trial (ISRCTN Registry ISRCTN67795930).

## Author contribution statement

SCM drafted the manuscript. CAM analysed the data. All authors reviewed and contributed to the final manuscript.
