# Supplementary material for: Subsequent pregnancy outcomes among women with tubal ectopic pregnancy treated with methotrexate
Source: Reprod Fertil. 2023 Jun 15;4(2):e230019. doi: 10.1530/RAF-23-0019 (PMC10305469; doi:10.1530/RAF-23-0019)
Supplement: Supplementary Table I. Pregnancy rates and subsequent pregnancy outcomes among women with tubal ectopic pregnancy medically, presented by GEM3 randomisation treatment (gefitinib and methotrexate versus placebo and methotrexate). [file supplementary_table_1.pdf]

Supplementary Table I. *Pregnancy rates and subsequent pregnancy outcomes among women with tubal ectopic pregnancy medically, presented by GEM3 randomisation treatment (gefitinib and methotrexate versus placebo and methotrexate).*

	Randomised treatment		p-value
	Gefitinib and methotrexate (N=165)	Placebo and methotrexate (N=162)	
Any pregnancy post-treatment- N (%)	76 (54)	73 (51)	0.68
Missing	24	20	
Any live birth post-treatment[1]-N (%)	52 (70)	41 (60)	0.21
Missing	2	5	
Any pregnancy loss post-treatment[1,2]-N (%)	24 (35)	31 (46)	0.17
Missing	7	6	
Any ectopic pregnancy post-treatment[1]-N (%)	10 (15)	12 (19)	0.56
Missing	9	9	

[1]Only in women whom have had a pregnancy post-treatment. [2]Defined as miscarriage, ectopic pregnancy, stillbirth or molar pregnancy (excluding termination of pregnancy).
